# Supplementary material for: The association between poverty and gene expression within peripheral blood mononuclear cells in a diverse Baltimore City cohort
Source: PLoS One. 2020 Sep 24;15(9):e0239654. doi: 10.1371/journal.pone.0239654 (PMC7514036; doi:10.1371/journal.pone.0239654)
Supplement: S1 Table — Data are presented as mean ± S.D. CRP: C-reactive protein; BP: blood pressure; HTN: hypertension. WBC: white blood cell count (count*10^9/L); monocytes: WBC monocyte count (10^9/L); dx: diagnosis); No significant difference of dxDiabetes (P>0.00), dxHTN (P = 0.572), or current smoking (P = 0.075) between below poverty and above using Fisher’s Exact Test. (PDF) [file pone.0239654.s001.pdf]

S1 Table: Demographics of the Microarray Cohort

Characteristic	African American Males Below Poverty (AAMBL) n= 7	African American Males Above Poverty (AAMAB) n= 6	White Males Below Poverty (WMBL) n= 6	White Males Above Poverty (WMAB) n= 7	White Females Below Poverty (WFBL) n= 6	White Females Above Poverty (WFAB) n= 7	African American Females Below Poverty (AAFBL) n= 7	African American Females Above Poverty (AAFAB) n= 6
Age, y	46.3 ± 9.12	50.2 ± 6.83	52.3 ± 5.64	50.0 ± 8.50	47.5 ± 4.87	47.1 ± 10.6	51.5 ± 8.32	51.3 ± 9.17
Total cholesterol, mg/dL	186 ± 26.6	173 ± 51.5	219 ± 43.5	172 ± 44.8	174 ± 27.8	200 ± 48.0	194 ± 28.8	182 ± 32.6
CRP, mg/L	1.45 ± 1.37	1.34 ± 1.57	4.15 ± 2.83	3.85 ± 2.22	1.66 ± 1.81	3.60 ± 3.01	4.47 ± 6.57	3.03 ± 3.56
Right Systolic BP, mmHg	123 ± 11.4	119 ± 10.2	107 ± 12.1	105 ± 13.1	113 ± 7.56	109 ± 11.2	129 ± 20.6	126 ± 12.7
Left Systolic BP, mmHg	123 ± 10.0	122 ± 10.0	105 ± 13.2	107 ± 14.8	111 ± 7.00	109 ± 11.5	128 ± 22.2	121 ± 16.1
Right Diastolic BP, mmHg	72.3 ± 11.2	70.3 ± 9.42	71.0 ± 12.0	66.3 ± 9.05	71.3 ± 4.50	65.1 ± 5.98	75.0 ± 13.5	74.3 ± 10.2
Left Diastolic BP, mmHg	73.7 ± 6.28	73.0 ± 8.24	67.7 ± 11.7	67.4 ± 10.5	72.3 ± 5.28	64.3 ± 6.26	77.0 ± 15.1	75.3 ± 10.3
Monocytes, n	107 ± 213	444 ± 381	154 ± 241	291± 172	340 ± 251	210 ± 298	495 ± 132	238 ± 282
WBC's, n	6.05 ± 2.39	5.23 ± 2.69	7.57 ± 1.47	6.03 ± 1.04	5.73 ± 1.89	6.98 ± 2.06	6.28 ± 1.60	5.77 ± 2.38
Diabetes	14.3 %	16.7%	33.3%	14.3 %	0.00%	0.00%	0.00%	16.7%
HTN	42.8%	16.7%	66.7%	42.8%	0.00%	28.6%	71.4%	50.0 %
Current Smoker	42.8%	50.0%	33.3%	14.3 %	66.7%	0.00%	42.8%	16.7%
