# Supplementary material for: The association between poverty and gene expression within peripheral blood mononuclear cells in a diverse Baltimore City cohort
Source: PLoS One. 2020 Sep 24;15(9):e0239654. doi: 10.1371/journal.pone.0239654 (PMC7514036; doi:10.1371/journal.pone.0239654)
Supplement: S2 Table — Data are presented as mean ± S.D. CRP: C-reactive protiein; BP: blood pressure; HTN: hypertension; WBC: white blood cell count (count*10^9/L); monocytes: WBC monocyte count (10^9/L); dx: diagnosis); No significant impact of dxDiabetes (P = 0.073), dxHTN (P = 0.37), or current smoking (P = 0.18) between below poverty vs. above using Fisher’s Exact Test. (PDF) [file pone.0239654.s002.pdf]

S2 Table: Demographics of the Validation Cohort

Characteristic	African American Male Below Poverty (AAMBL) n= 27	African American Male Above Poverty (AAMAB) n= 29
Age, y	44.8 ± 9.11	47.8 ± 8.01
Total cholesterol, mg/dL	173 ± 39.2	178 ± 36.3
CRP, mg/L	2.71 ± 3.46	6.07 ± 16.2
Right Systolic BP, mmHg	121 ± 14.2	119 ± 12.4
Left Systolic BP, mmHg	120 ± 17.0	120 ± 12.7
Right Diastolic BP, mmHg	73.8 ± 12.6	73.0 ± 11.0
Left Diastolic BP, mmHg	73.9 ± 12.6	74.4 ± 10.6
Monocytes, n	268 ± 328	292 ± 237
WBC's, n	6.59 ± 3.17	6.01 ± 1.87
dxDiabetes	25.9 % (7/27)	6.89 % (2/29)
dxHTN	33.3 % (9/27)	20.7 % (6/29)
Current Smoker	59.3 % (16/27)	37.9 % (11/29)
