# Supplementary material for: The association between poverty and gene expression within peripheral blood mononuclear cells in a diverse Baltimore City cohort
Source: PLoS One. 2020 Sep 24;15(9):e0239654. doi: 10.1371/journal.pone.0239654 (PMC7514036; doi:10.1371/journal.pone.0239654)
Supplement: S3 Table — (PDF) [file pone.0239654.s003.pdf]

S3 Table: RT-qPCR Primer Sequences.

**Gene**	**Forward**	**Reverse**
CD19	CCCTGGGGTCCCAGTCCTAT	TTCCTCATGATTGGGTCCAGG
CD36	GAGGACTGCAGTGTAGGACTT	TGGCTAAGAAGGATTTTTCAATCA
DUSP2	TACTTCCTGCGAGGAGGCTT	TGGTTTTGTCCCCTGTTGGC
GIMAP1	ACGTGAGCAACACAGAGAAC	CCGGTTATCAAAGGCACAGAC
HLA-DQB1	TGCTACTTCACCAACGGGAC	TCGAAGCGCACGATCTCT
KCTD12	GTGCGTGGGACAGTAAAGGA	GGCAGGCGGATCACTTTCTA
KLF6	CCACTTGAAAGCACACCAGC	CTTGCAAAACGCCACTCACA
RBM38	GCTACGGCTTCGTGACCAT	GATGATGGGGTTCGGGTCTTT
GAPDH	GCTCCTCCTGTTCGACAGTCA	ACCTTCCCCATGGTGTCTGA
ACTB	GGACTTCGAGCAAGAGATGG	AGCACTGTGTTGGCGTACAG
