# Supplementary material for: The association between poverty and gene expression within peripheral blood mononuclear cells in a diverse Baltimore City cohort
Source: PLoS One. 2020 Sep 24;15(9):e0239654. doi: 10.1371/journal.pone.0239654 (PMC7514036; doi:10.1371/journal.pone.0239654)

S1 Figure. Gene ontology and pathway analysis of top sex-related (A) or race-related (B) differentially-expressed genes

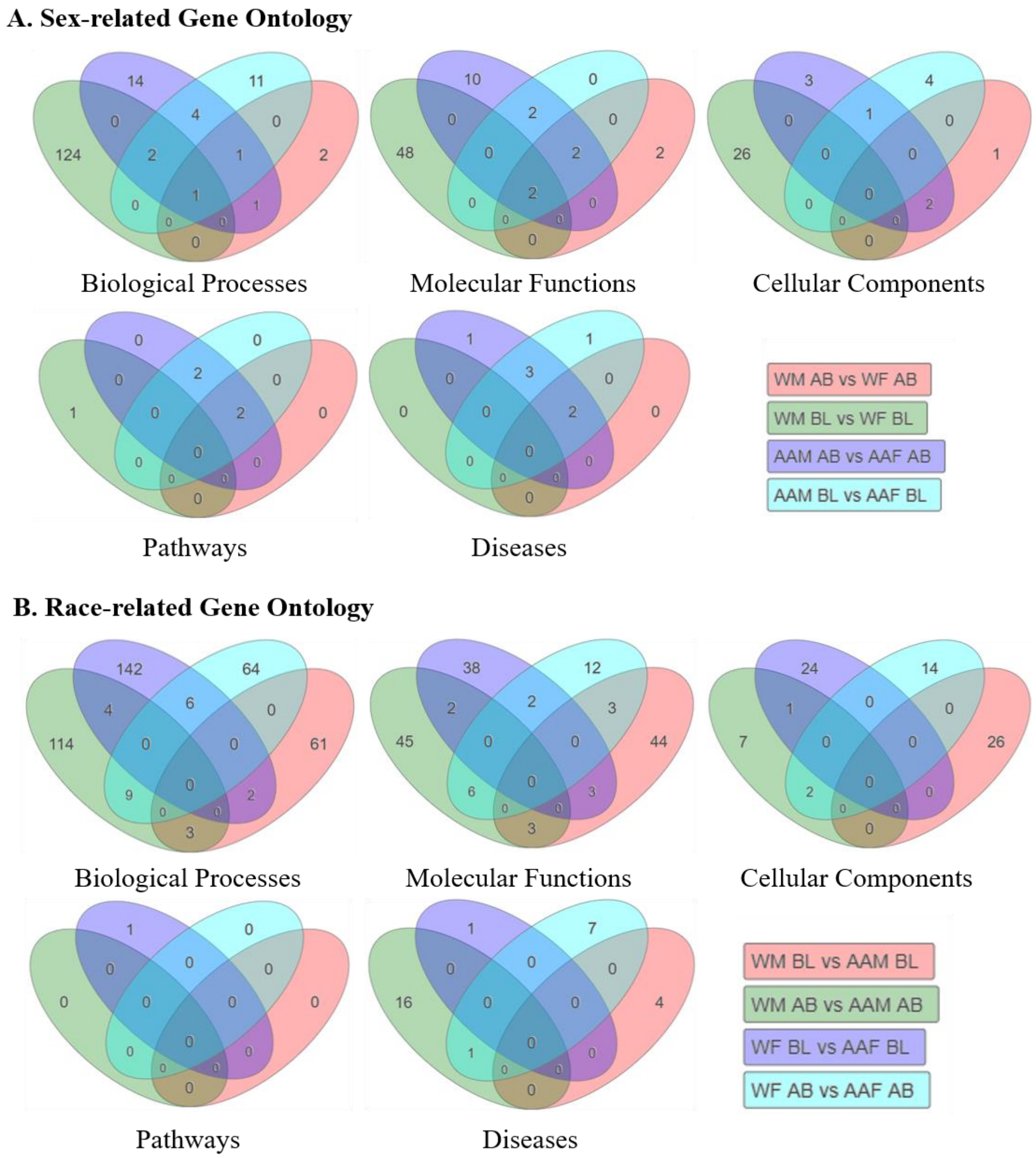

Supplement: S1 Fig — Gene ontology and pathway analysis of top sex-related (A) or race-related (B) differentially-expressed genes. Venn diagrams break down significant GO terms related to Biological Processes, Molecular Function, or Cellular Components and stratified by sex (B) or ancestry (C) for each comparison. iPathway Guide identification of unique and overlapping genetic pathways or diseases related for each comparison. N = 6/7 per group; see S1 Table for completely demographics. White females below poverty (WFBL) or above poverty (WFAB); white males below poverty (WMBL) or above (WMAB); African American males below poverty (AAMBL) or above (AAMAB); African American females below poverty (AAFBL) or above (AAFAB). (PDF) [file pone.0239654.s004.pdf]
