# Supplementary figures and images for: The association between poverty and gene expression within peripheral blood mononuclear cells in a diverse Baltimore City cohort
Source: PLoS One. 2020 Sep 24;15(9):e0239654. doi: 10.1371/journal.pone.0239654 (PMC7514036; doi:10.1371/journal.pone.0239654)

S2 Figure. Gene ontology (GO) molecular function analysis in African American females and males.

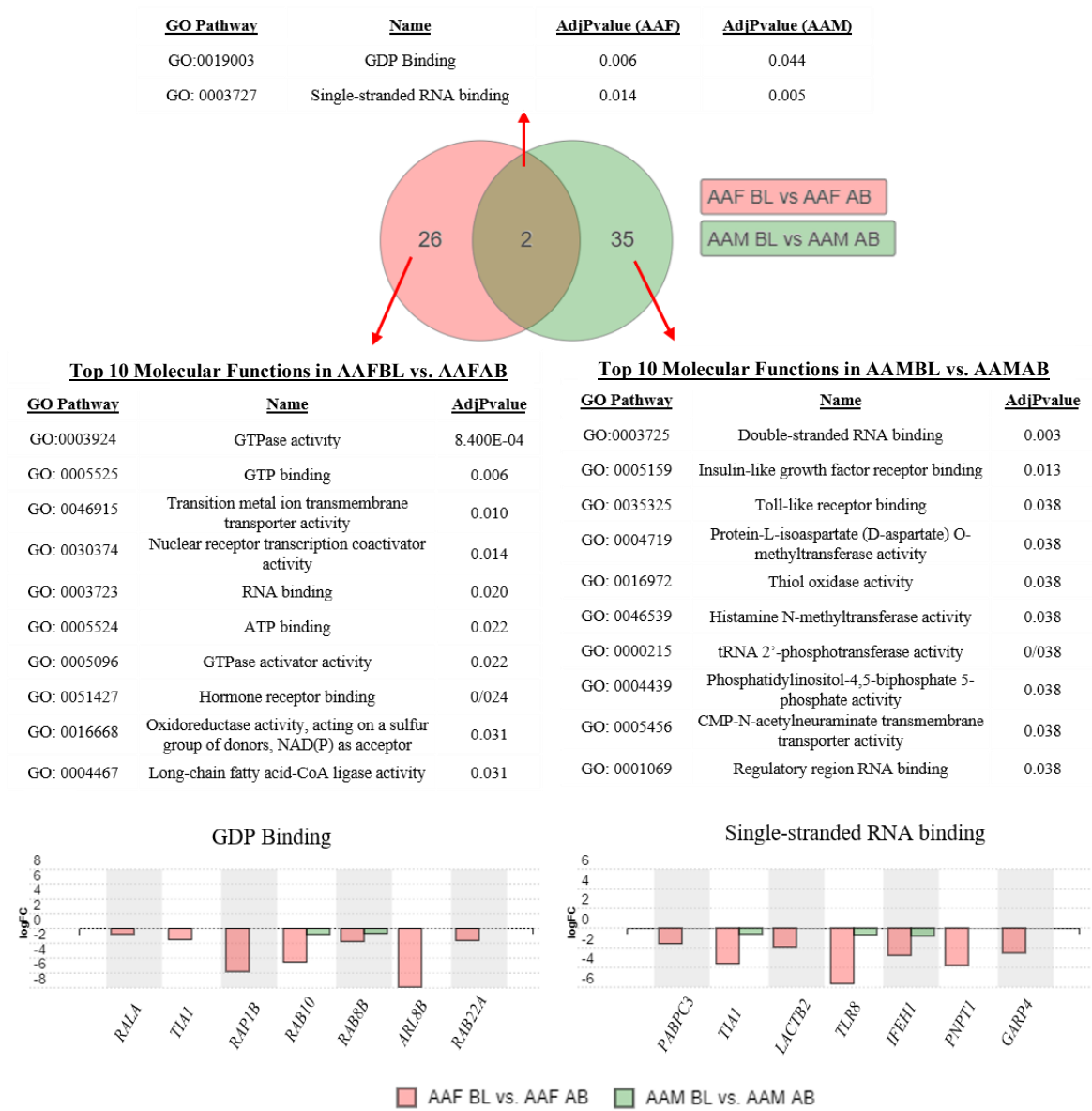

Supplement: S2 Fig — (A) Venn diagram of GO Molecular Functions of significant genes identified in AAFBL vs. AAFAB compared with AAMBL vs. AAMAB. Unique and common pathways for each comparison of poverty status in females (pink) and males (green) were identified after elimination pruning P-value adjustment to eliminate false positives. Tables provide Top 10 Molecular Functions unique to AA females or AA males or overlapping between both groups. Pathways are reported with GO ascension number, name, and adjusted P-value (AdjPvalue). (B) Differential expression of significant genes identified common in the two overlapping pathways between AA females and males. Gene expression is reported as logFC in AAFBL vs. AAFAB (pink) or AAMBL vs. AAMAB (green). (PDF) [file pone.0239654.s005.pdf]

S3 Figure. Gene ontology (GO) cellular components analysis in African American females and males.

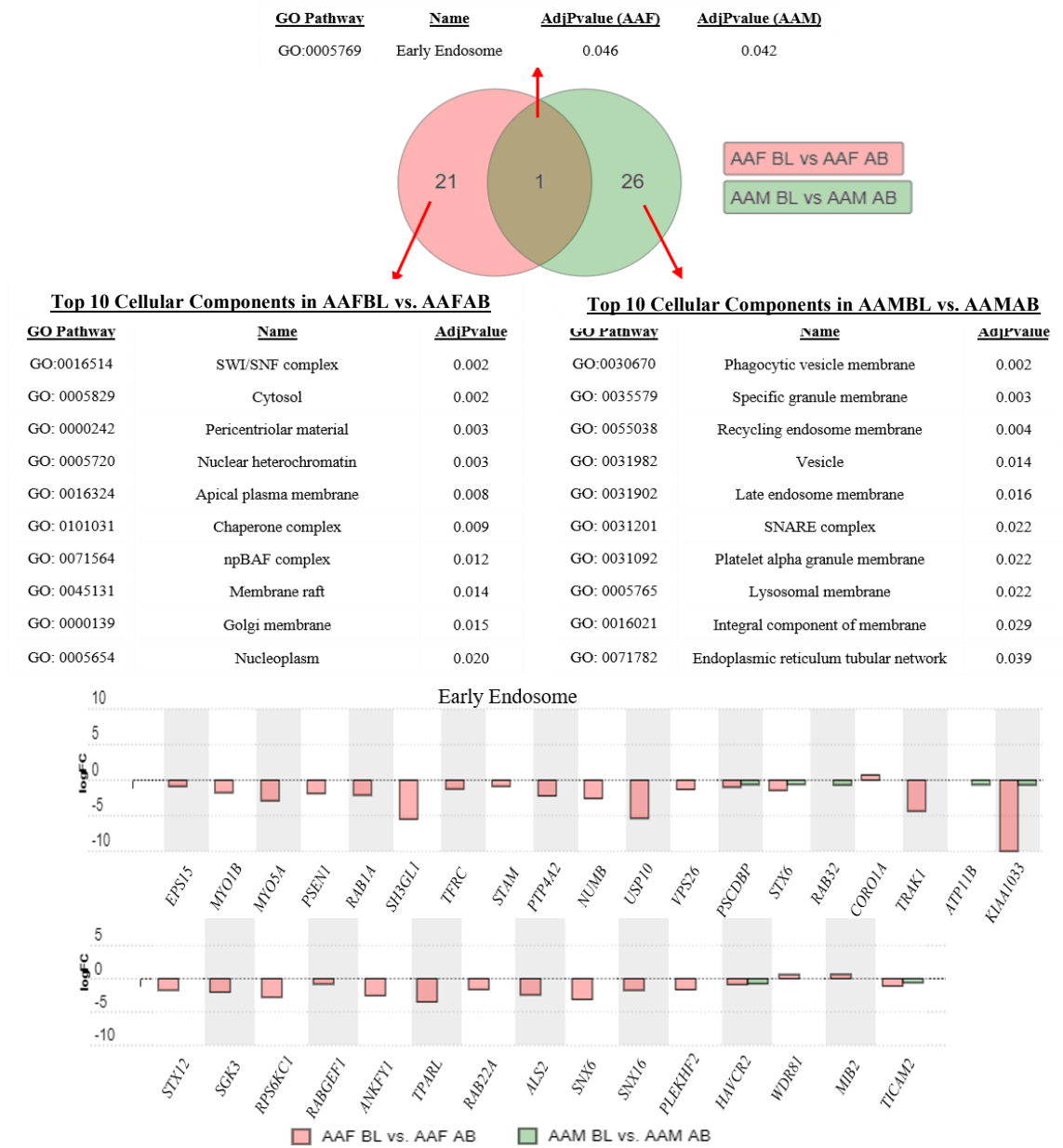

Supplement: S3 Fig — (A) Venn diagram of GO Cellular Components of significant genes identified in AAFBL vs. AAFAB compared with AAMBL vs. AAMAB. Unique and common pathways for each comparison of poverty status in females (pink) and males (green) were identified after elimination pruning P-value adjustment to eliminate false positives. Tables provide Top 10 Cellular Components unique to AA females or AA males or overlapping between both groups. Pathways are reported with GO ascension number, name, and adjusted P-value (AdjPvalue). (B) Differential expression of significant genes identified common in the two overlapping pathways between AA females and males. Gene expression is reported as logFC in AAFBL vs. AAFAB (pink) or AAMBL vs. AAMAB (green). (PDF) [file pone.0239654.s006.pdf]
