# Supplementary material for: The association between poverty and gene expression within peripheral blood mononuclear cells in a diverse Baltimore City cohort
Source: PLoS One. 2020 Sep 24;15(9):e0239654. doi: 10.1371/journal.pone.0239654 (PMC7514036; doi:10.1371/journal.pone.0239654)
Supplement: S1 Appendix — (DOC) [file pone.0239654.s009.doc]

**The Association between Poverty and Gene Expression within Peripheral Blood Mononuclear Cells in a Diverse Baltimore City Cohort**

Nicole S. Arnold^1^, Nicole Noren Hooten^2^, Yongqing Zhang^3^, Elin Lehrmann^3^, William Wood III^3^, Wendy Camejo Nunez^1^, Roland J. Thorpe^4^, Michele K. Evans^2^, and Douglas F. Dluzen^1*^

^1^Department of Biology, Morgan State University, Baltimore, MD

^2^Laboratory of Epidemiology and Population Science, National Institute on Aging, National Institutes of Health, Baltimore, MD

^3^Laboratory of Genetics, National Institute on Aging, National Institutes of Health, Baltimore, MD

^4^Program for Research on Men’s Health, Hopkins Center for Health Disparities Solutions, Johns Hopkins University, Baltimore, MD

Correspondence:

*douglas.dluzen@morgan.edu

**Supplemental Information**

**Supplemental Table 1: Demographics of the Microarrry Cohort**

| **Characteristic** | **African American Males Below Poverty (AAMBL) n= 7** | **African American Males Above Poverty (AAMAB) n= 6** | **White Males Below Poverty (WMBL) n= 6** | **White Males Above Poverty (WMAB) n= 7** | **White Females Below Poverty (WFBL) n= 6** | **White Females Above Poverty (WFAB) n= 7** | **African American Females Below Poverty (AAFBL) n= 7** | **African American Females Above Poverty (AAFAB) n= 6** |
| --- | --- | --- | --- | --- | --- | --- | --- | --- |
|
| Age, y | 46.3 ± 9.12 | 50.2 ± 6.83 | 52.3 ± 5.64 | 50.0 ± 8.50 | 47.5 ± 4.87 | 47.1 ± 10.6 | 51.5 ± 8.32 | 51.3 ± 9.17 |
| Total cholesterol, mg/dL | 186 ± 26.6 | 173 ± 51.5 | 219 ± 43.5 | 172 ± 44.8 | 174 ± 27.8 | 200 ± 48.0 | 194 ± 28.8 | 182 ± 32.6 |
| CRP, mg/L | 1.45 ± 1.37 | 1.34 ± 1.57 | 4.15 ± 2.83 | 3.85 ± 2.22 | 1.66 ± 1.81 | 3.60 ± 3.01 | 4.47 ± 6.57 | 3.03 ± 3.56 |
| Right Systolic BP, mmHg | 123 ± 11.4 | 119 ± 10.2 | 107 ± 12.1 | 105 ± 13.1 | 113 ± 7.56 | 109 ± 11.2 | 129 ± 20.6 | 126 ± 12.7 |
| Left Systolic BP, mmHg | 123 ± 10.0 | 122 ± 10.0 | 105 ± 13.2 | 107 ± 14.8 | 111 ± 7.00 | 109 ± 11.5 | 128 ± 22.2 | 121 ± 16.1 |
| Right Diastolic BP, mmHg | 72.3 ± 11.2 | 70.3 ± 9.42 | 71.0 ± 12.0 | 66.3 ± 9.05 | 71.3 ± 4.50 | 65.1 ± 5.98 | 75.0 ± 13.5 | 74.3 ± 10.2 |
|
| Left Diastolic BP, mmHg | 73.7 ± 6.28 | 73.0 ± 8.24 | 67.7 ± 11.7 | 67.4 ± 10.5 | 72.3 ± 5.28 | 64.3 ± 6.26 | 77.0 ± 15.1 | 75.3 ± 10.3 |
|
| Monocytes, n | 107 ± 213 | 444 ± 381 | 154 ± 241 | 291± 172 | 340 ± 251 | 210 ± 298 | 495 ± 132 | 238 ± 282 |
|
| WBC's, n | 6.05 ± 2.39 | 5.23 ± 2.69 | 7.57 ± 1.47 | 6.03 ± 1.04 | 5.73 ± 1.89 | 6.98 ± 2.06 | 6.28 ± 1.60 | 5.77 ± 2.38 |
|
| Diabetes | 14.3 % | 16.7% | 33.3% | 14.3 % | 0.00% | 0.00% | 0.00% | 16.7% |
| HTN | 42.8% | 16.7% | 66.7% | 42.8% | 0.00% | 28.6% | 71.4% | 50.0 % |
| Current Smoker | 42.8% | 50.0% | 33.3% | 14.3 % | 66.7% | 0.00% | 42.8% | 16.7% |
| **Data are presented as mean ± S.D**. CRP: C-reactive protein; BP: blood pressure; HTN: hypertension. WBC: white blood cell count (count*10^9/L); monocytes: WBC monocyte count (10^9/L); dx: diagnosis); No significant difference of dxDiabetes (P>0.00), dxHTN (P=0.572), or current smoking (P=0.075) between below poverty and above using Fisher’s Exact Test. | | | | | | | | |

**Supplemental Table 2: Demographics of the Validation Cohort**

| **Characteristic** | **African American Male Below Poverty (AAMBL) n= 27** | **African American Male Above Poverty (AAMAB) n= 29** |
| --- | --- | --- |
|
| Age, y | 44.8 ± 9.11 | 47.8 ± 8.01 |
| Total cholesterol, mg/dL | 173 ± 39.2 | 178 ± 36.3 |
| CRP, mg/L | 2.71 ± 3.46 | 6.07 ± 16.2 |
| Right Systolic BP, mmHg | 121 ± 14.2 | 119 ± 12.4 |
| Left Systolic BP, mmHg | 120 ± 17.0 | 120 ± 12.7 |
| Right Diastolic BP, mmHg | 73.8 ± 12.6 | 73.0 ± 11.0 |
|
| Left Diastolic BP, mmHg | 73.9 ± 12.6 | 74.4 ± 10.6 |
|
| Monocytes, n | 268 ± 328 | 292 ± 237 |
|
| WBC's, n | 6.59 ± 3.17 | 6.01 ± 1.87 |
|
| dxDiabetes | 25.9 % (7/27) | 6.89 % (2/29) |
| dxHTN | 33.3 % (9/27) | 20.7 % (6/29) |
| Current Smoker | 59.3 % (16/27) | 37.9 % (11/29) |
| **Data are presented as mean ± S.D**. CRP: C-reactive protiein; BP: blood pressure; HTN: hypertension; WBC: white blood cell count (count*10^9/L); monocytes: WBC monocyte count (10^9/L); dx: diagnosis); No significant impact of dxDiabetes (P=0.073), dxHTN (P=0.37), or current smoking (P=0.18) between below poverty vs. above using Fisher’s Exact Test | | |

**Supplemental Table 3: RT-qPCR Primer**

| **Gene** | **Forward** | **Reverse** |
| --- | --- | --- |
|
| *CD19* | CCCTGGGGTCCCAGTCCTAT | TTCCTCATGATTGGGTCCAGG |
| *CD36* | GAGGACTGCAGTGTAGGACTT | TGGCTAAGAAGGATTTTTCAATCA |
| *DUSP2* | TACTTCCTGCGAGGAGGCTT | TGGTTTTGTCCCCTGTTGGC |
| *GIMAP1*  *HLA-DQB1* | ACGTGAGCAACACAGAGAAC  TGCTACTTCACCAACGGGAC | CCGGTTATCAAAGGCACAGAC  TCGAAGCGCACGATCTCT |
|
| *KCTD12*  *KLF6* | GTGCGTGGGACAGTAAAGGA  CCACTTGAAAGCACACCAGC | GGCAGGCGGATCACTTTCTA  CTTGCAAAACGCCACTCACA |
|
| *RBM38*  *GAPDH*  *ACTB* | GCTACGGCTTCGTGACCAT  GCTCCTCCTGTTCGACAGTCA  GGACTTCGAGCAAGAGATGG | GATGATGGGGTTCGGGTCTTT  ACCTTCCCCATGGTGTCTGA  AGCACTGTGTTGGCGTACAG |
|
|


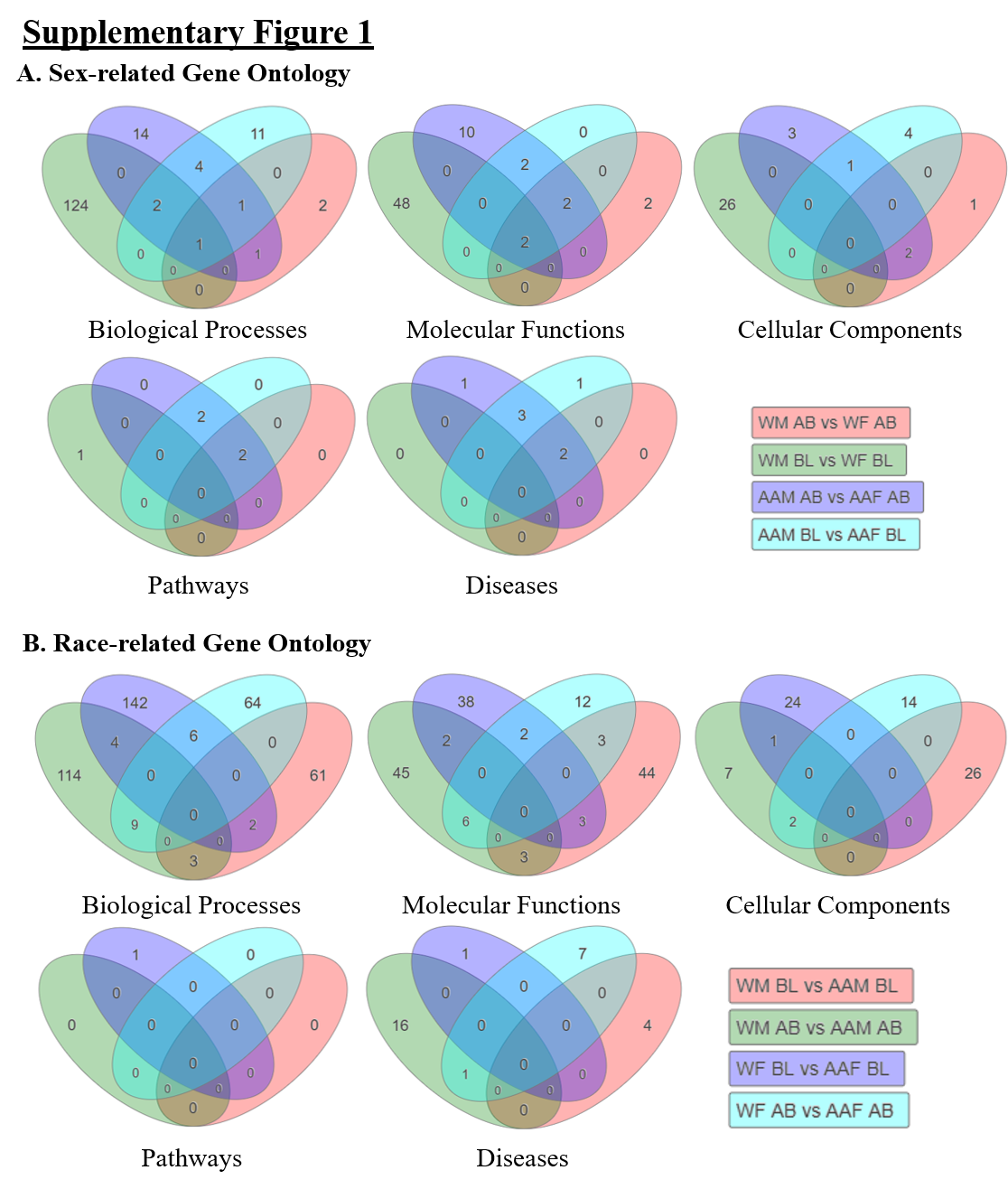


**Supplementary Figure 1: Gene ontology and pathway analysis of top sex-related (A) or race-related (B) differentially-expressed genes.** Venn diagrams break down significant GO terms related to Biological Processes, Molecular Function, or Cellular Components and stratified by sex (B) or ancestry (C) for each comparison. iPathway Guide identification of unique and overlapping genetic pathways or diseases related for each comparison. N= 6/7 per group; see Suppl. Table 1 for completely demographics. White females below poverty (WFBL) or above poverty (WFAB); white males below poverty (WMBL) or above (WMAB); African American males below poverty (AAMBL) or above (AAMAB); African American females below poverty (AAFBL) or above (AAFAB).


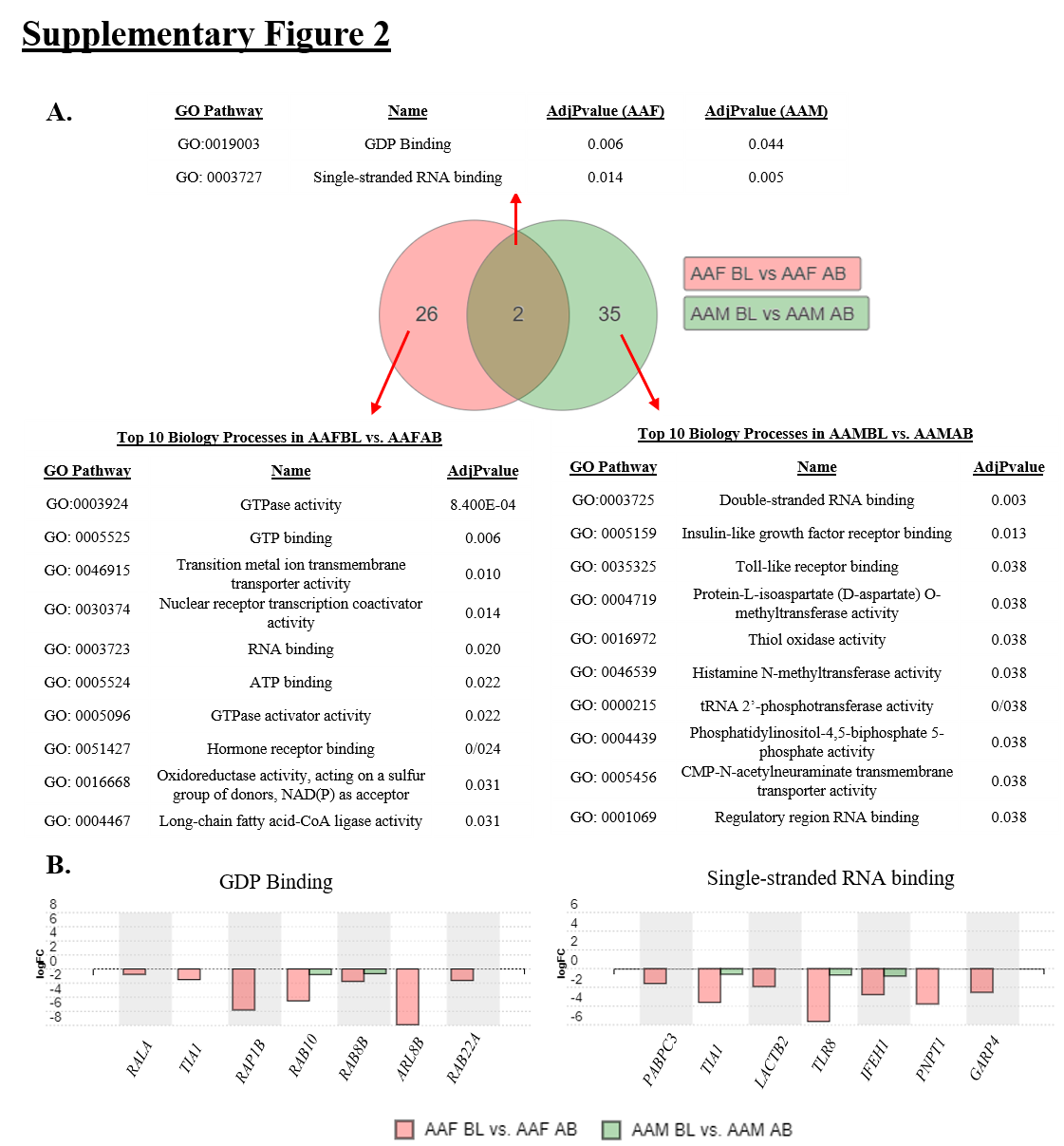


**Supplementary Figure 2: Gene ontology (GO) analysis in African American females and males.** (A) Venn diagram of GO Molecular Functions of significant genes identified in AAFBL vs. AAFAB compared with AAMBL vs. AAMAB. Unique and common pathways for each comparison of poverty status in females (pink) and males (green) were identified after elimination pruning P-value adjustment to eliminate false positives. Tables provide Top 10 Molecular Functions unique to AA females or AA males or overlapping between both groups. Pathways are reported with GO ascension number, name, and adjusted P-value (AdjPvalue). (B) Differential expression of significant genes identified common in the two overlapping pathways between AA females and males. Gene expression is reported as logFC in AAFBL vs. AAFAB (pink) or AAMBL vs. AAMAB (green).


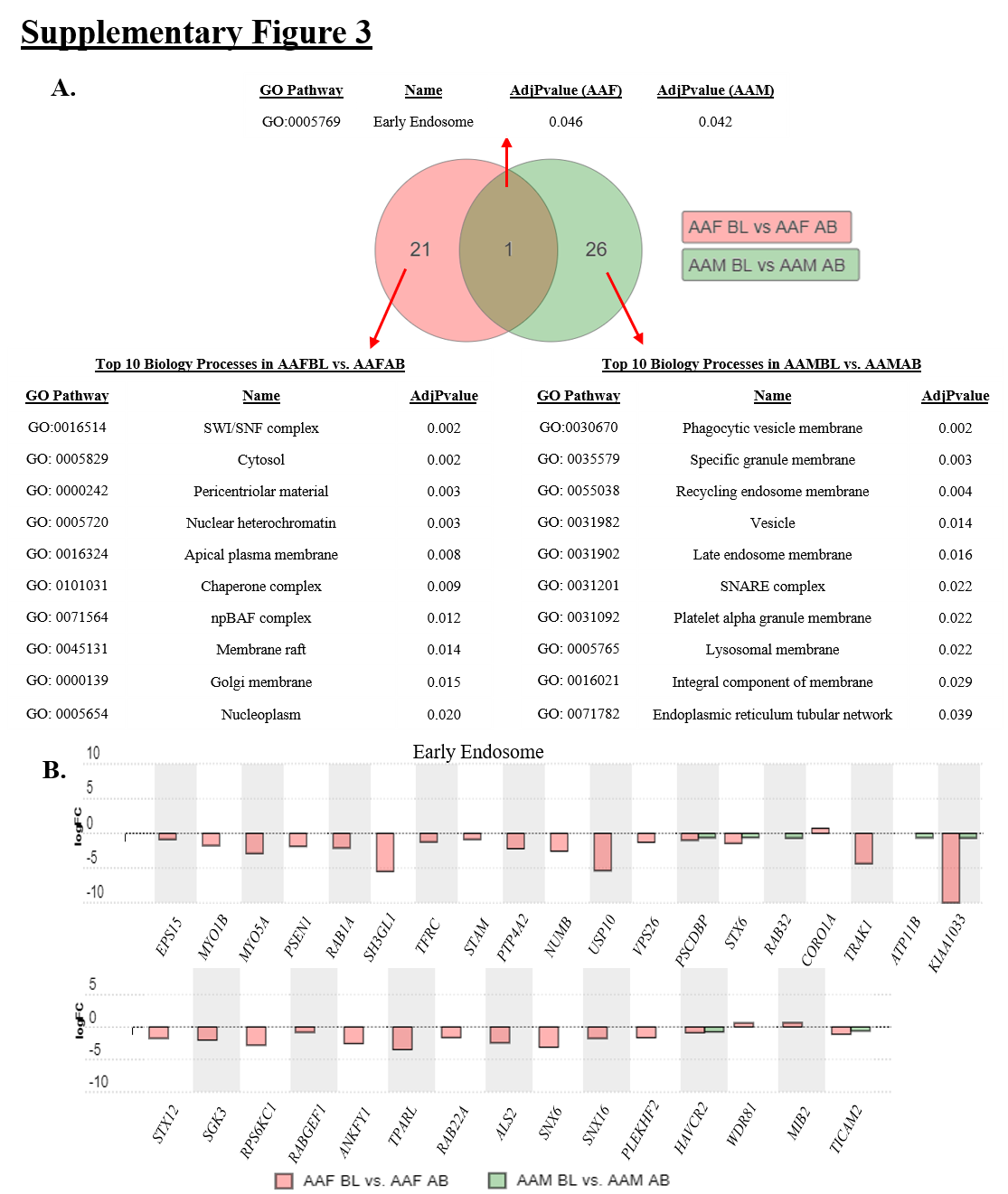


**Supplementary Figure 3: Gene ontology (GO) analysis in African American females and males.** (A) Venn diagram of GO Cellular Components of significant genes identified in AAFBL vs. AAFAB compared with AAMBL vs. AAMAB. Unique and common pathways for each comparison of poverty status in females (pink) and males (green) were identified after elimination pruning P-value adjustment to eliminate false positives. Tables provide Top 10 Cellular Components unique to AA females or AA males or overlapping between both groups. Pathways are reported with GO ascension number, name, and adjusted P-value (AdjPvalue). (B) Differential expression of significant genes identified common in the two overlapping pathways between AA females and males. Gene expression is reported as logFC in AAFBL vs. AAFAB (pink) or AAMBL vs. AAMAB (green).
